# Effect of Five Polymorphisms on Percentage of Oleic Acid in Beef and Investigation of Linkage Disequilibrium to Confirm the Locations of Quantitative Trait Loci on BTA19 in Japanese Black Cattle

**DOI:** 10.3390/life11070597

**Published:** 2021-06-22

**Authors:** Fuki Kawaguchi, Fuka Kakiuchi, Kenji Oyama, Hideyuki Mannen, Shinji Sasazaki

**Affiliations:** 1 Laboratory of Animal Breeding and Genetics, Graduate School of Agricultural Science, Kobe University, Kobe 657-8501, Japan; kawaguchi@koala.kobe-u.ac.jp (F.K.); 1816127a@stu.kobe-u.ac.jp (F.K.); mannen@kobe-u.ac.jp (H.M.); 2 Food Resources Education & Research Center, Kobe University, Kasai 675-2103, Japan; oyama@kobe-u.ac.jp

**Keywords:** quantitative trait loci, fatty acid composition, Japanese Black cattle

## Abstract

Five polymorphisms associated with the percentage of oleic acid (C18:1) in beef fat were previously reported on bovine chromosome 19 in different Japanese Black cattle populations. This study aimed to verify the effects of these five polymorphisms on C18:1 using the same Japanese Black cattle population and conduct linkage disequilibrium (LD) analysis in order to determine the locations of the quantitative trait loci (QTLs). We genotyped the five polymorphisms (*SREBP1* c.1065 + 83 (84bp indel), *STARD3* c.1187 C > T, *GH* c.379 C > G, *FASN* g.841 G > C, and *FASN* g.16024 A > G) in two populations, which were bred in Hyogo and Gifu Prefectures, Japan (n = 441 and 443, respectively) in order to analyze their effects on C18:1 using analysis of variance (ANOVA). In the Hyogo population, *SREBP1* c.1065 + 83 and *STARD3* c.1187 C > T were significantly associated with C18:1 (*p* < 0.001). Meanwhile, *FASN* g.841 G > C, *FASN* g.16024 A > G, and *GH* c.379 C > G were significantly associated with C18:1 (*p* < 0.01) in the Gifu population. LD analysis was subsequently conducted to detect the range of the QTLs, which ranged from 32.2 to 46.4 Mbp and from 47.8 to 52.1 Mbp in the Hyogo and Gifu populations, respectively. In conclusion, this study confirmed the existence of QTLs on BTA19 and divided the candidate region for each QTL based on LD coefficients. These results could contribute to efficient searches for responsible genes and polymorphisms for fatty acid composition.

## 1. Introduction

Fat quality has been an important indicator of beef quality in the past few decades. In particular, a percentage of fatty acids, known as the fatty acid composition, is regarded worldwide as essential for beef flavor and tenderness. A high percentage of unsaturated fatty acids (UFAs) exhibits a positive impact on beef tenderness due to the lower melting point of UFAs [1]. Additionally, UFAs in phospholipids exhibit an important role in flavor development [2]. Therefore, beef with a high percentage of UFAs is evaluated as being of high quality. Oleic acid (C18:1) usually forms the highest percentage of all UFAs included in beef [2]. Therefore, the percentage of C18:1 is one of the factors that determines the palatability of beef and is an important indicator of beef quality.

In Japanese Black cattle, some researchers reported associations between fatty acid composition and polymorphisms within genes. Interestingly, some of the polymorphisms are dominantly localized in the region from 35 to 52 Mbp on BTA19. Among them, two single-nucleotide polymorphisms (SNPs) within the *FASN* gene (g.841 G > C and g.16024 A > G), which are located on about 51.4 Mbp, are well known for being polymorphisms that are likely responsible for fatty acid composition because of associations between the polymorphisms and fatty acid composition in various cattle populations [3,4,5,6]. Additionally, three polymorphisms within the *SREBP1*, *STARD3*, and *GH* genes have also been reported to be significantly associated with fatty acid composition [7,8,9]. These three polymorphisms, *SREBP1* c.1065 + 83, *STARD3* c.1187 C > T, and *GH* c.379 C > G, were located on about 35.2, 40.7, and 48.6 Mbp on BTA19, respectively.

Previous studies have verified the possibility that these polymorphisms are responsible for fatty acid composition. Among them, *FASN* polymorphisms were reported as the most likely candidates, principally in terms of *FASN* gene function and the impacts of polymorphisms on gene structure. The *FASN* gene demonstrates a role in the synthesis of fatty acids, especially palmitate (C16:0) [10]. As an increase in C16:0 content in beef can cause a decrease in the percentage of C18:1, the *FASN* gene could be a promising candidate. Additionally, researchers suggested that *FASN* polymorphisms either alter the spatial structure of the β-ketoacyl reductase domain, which is important for *FASN* activity, [3] or change the expression level of *FASN* gene by the alteration of binding activity of the transcription factor Sp1 [11]. Hence, *FASN* polymorphisms could affect the percentage of C18:1 in beef by changing the function of the *FASN* gene.

However, *SREBP1*, *STARD3*, and *GH* polymorphisms are not that plausible as responsible polymorphisms for fatty acid composition. The *SREBP1* gene encodes a transcription factor for genes involved in fatty acid metabolism, such as *Stearoyl-CoA desaturase* (*SCD*), which regulates fatty acid desaturation [12]. A previous study by Gamarra et al. [13] reported that the expression level of the *SREBP1* gene was correlated with the expression level of the *SCD* gene and the content of each fatty acid. However, the study also showed that the genotype of *SREBP1* c.1065 + 83 was not significantly associated with the expression level of the *SREBP1* gene. Therefore, this study concluded that further study is needed to elucidate the effect of the indel on the function of the *SREBP1* gene. Meanwhile, the *STARD3* gene encodes a transmembrane protein, which exhibits a role in the maintenance of cholesterol distribution and homeostasis [14,15], and the *GH* gene encodes a peptide hormone to enhance muscle growth and regulate adipose tissue in cattle [16,17]. Although they are certainly related to fat metabolism, the functional relationships between these genes and fatty acid composition remain unclear.

As described above, five polymorphisms that were significantly associated with fatty acid composition were identified on BTA19 in Japanese Black cattle in previous studies. Among them, *FASN* polymorphisms are likely to be responsible for fatty acid composition and could be effective markers for selection. However, the associations between fatty acid composition and the other polymorphisms within the three genes imply the existence of other responsible polymorphisms near the *FASN* polymorphisms. Alternatively, the association could be caused by high linkage disequilibrium (LD) between the *FASN* polymorphisms and the other three polymorphisms. Conducting LD analysis between these five polymorphisms could provide some evidence to clarify this possibility. However, doing this has not yet been possible because the previous studies about these five polymorphisms were conducted in different populations. Therefore, this investigation started to try to estimate the effect of the five polymorphisms on the percentage of C18:1 in beef by using the same population and analyzing LD to verify the position of their quantitative trait loci (QTLs) on BTA19.

## 2. Materials and Methods

Two Japanese Black cattle populations (“Hyogo population” and “Gifu population”) were used to verify the effects of the five polymorphisms for fatty acid composition on BTA19 (Table 1). Since Japanese Black cattle has been managed under the different breeding systems according to the prefecture, the genetic background for the populations depends on the history of breeding in the prefecture in which they have been bred. The Hyogo population comprised the commercial cattle produced in Hyogo Prefecture, Japan from 2010 to 2011 and included 441 cattle (352 steers and 89 heifers). Similarly, the Gifu population comprised the commercial cattle produced in Gifu Prefecture, Japan from 2007 to 2009 and included 443 cattle which were all steers. The average ages at slaughter in the Hyogo and Gifu populations were 31.69 ± 1.24 and 28.32 ± 1.10 months, respectively. DNA was extracted from either the longissimus cervicis or thoracic muscle of these animals using the standard phenol–chloroform method [18].

Intramuscular fat tissue samples were collected from the longissimus thoracis muscle in both populations. Total lipid extraction was performed in order to analyze the percentage of C18:1 in the fatty acid of beef samples following a previous study [19]. The extracted fat was saponified with potassium hydrate–ethanol solution and then methyl-esterified with a boron trifluoride–methanol complex. Then, the processed fat was analyzed using gas chromatography (6890A; Agilent Technologies, Santa Clara, CA, USA) under the following conditions: the temperature of the inlet was 150 °C, the oven was warmed from 150 °C to 220 °C, and the temperature of the detector sensor was 220 °C. Helium gas was used as a carrier, a capillary column (TC-70, 0.25 mm I.D. × 60 m, df = 0.25 μm; GL Science, Tokyo, Japan), and a flame ionization detector for detection. The C18:1 content of the beef was finally expressed as a percentage of the total fatty acid content. The average percentage of C18:1 in the Hyogo and Gifu populations was 54.76% ± 3.01% and 51.92% ± 3.29%, respectively.

The current study was focused on five polymorphisms (Table 2). Among them, *SREBP1* c.1065 + 83 (84bp indel), *STARD3* c.1187 C > T, and *FASN* g.16024 A > G were genotyped in the Hyogo population in previous studies [8,20]. Additionally, *FASN* g.16024 A > G was also previously genotyped in the Gifu population [5]. The genotyping results were obtained for each animal from these previous studies. Additionally, genotyped *FASN* g.841 G > C was obtained in both populations and *SREBP1* c.1065 + 83 and *STARD3* c.1187 C > T in the Gifu population using the method described in the previous studies [5,7,8]. *GH* c.379 C > G was additionally genotyped using restriction fragment length polymorphism of fragments amplified by polymerase chain reaction and TaqMan hydrolysis probes in the Hyogo and Gifu populations. The primer and probe sequences, reaction conditions, and restriction enzymes are shown in Table 3.

Illumina BovineSNP50 v3 BeadChip (Illumina, San Diego, CA, USA) was applied to 200 randomly selected animals from the Hyogo population. Illumina BovineSNP50 v2 BeadChip (Illumina, San Diego, CA, USA) was applied to all animals in the Gifu population in a previous study [22]. Image data were analyzed using the Chromosome Viewer tool in BEADSTUDIO (Illumina) software. In this study, the genotype data of SNPs on BTA19 were used to calculate LD coefficients (*r*^2^). The set of SNPs was named as “50K SNPs.”

The effects of the five gene markers on the percentage of C18:1 were statistically tested using analysis of variance (ANOVA) and an appropriate model for each population. The model included the effect of genotype, slaughter month, sex, sire, and linear and quadratic covariates for the age at slaughter. However, the effect of slaughter month, sex, and sire were reduced from the model in Gifu population since they were not statistically significant.

A total of 1420 and 1391 SNPs were located on BTA19 in Illumina BovineSNP50 v2 BeadChip and Illumina BovineSNP50 v3 BeadChip, respectively. Among the 50K SNPs, 201 and 439 SNPs were excluded since their alleles were completely fixed in each population used in this study. Finally, 1219 and 952 SNPs were used to conduct LD analysis in Gifu and Hyogo populations, respectively. The LD coefficients (*r*^2^) between polymorphisms were calculated using HAPLOVIEW 4.0 with default settings.

## 3. Results

### 3.1. Genotype and Allele Frequency

Five gene polymorphisms were genotyped in the Hyogo and Gifu populations (Table 4 and Table 5). In the Hyogo population, the minor allele frequencies of *SREBP1* c.1065 + 83, *STARD3* c.1187 C > T, and *FASN* g.16024 A > G were 0.21, 0.45, and 0.10, respectively. *GH* c.379 C > G and *FASN* g.841 G > C were genotyped for only about 50 animals, since it became clear that their alleles were almost fixed according to their genotyping results. These two markers were excluded from the statistical analysis. In the Gifu population, all five markers were polymorphic, and the minor allele frequencies ranged from 0.09 to 0.49.

### 3.2. Association between C18:1 and Gene Markers

Table 4 and Table 5 show the associations between the five gene markers and the percentage of C18:1 based on ANOVA. *SREBP1* and *STARD3* polymorphisms were significantly associated with the percentage of C18:1 in the Hyogo population (*p* < 0.001), while *FASN* g.16024 A > G was not (*p* = 0.614). In contrast, *FASN* g.16024 A > G showed a significant association in the Gifu population (*p* = 0.001). Moreover, *GH* c.379 C > G and *FASN* g.841 G > C were also significantly associated in the Gifu population (*p* < 0.01).

### 3.3. LD among Five Gene Polymorphisms

**Figure 1:**
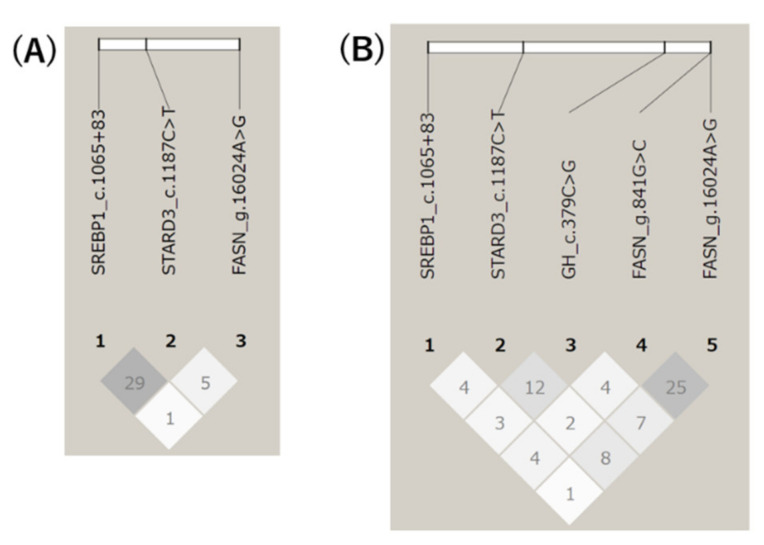
Linkage disequilibrium among the polymorphisms in Hyogo (**A**) and Gifu (**B**) populations. The *r*^2^ values are shown in each box with darker color representing stronger LD.

In the Hyogo population, the LD coefficient (*r*^2^) between *SREBP1* c.1065 + 83 and *STARD3* c.1187 C > T showed a medium level of LD (*r*^2^ = 0.29; Figure 1A). However, *FASN* g.16024 A > G showed an extremely low level of LD with the other two markers (*r*^2^ = 0.01 and 0.05). In the Gifu population, the coefficient among all five markers was calculated (Figure 1B). Two markers on the *FASN* gene showed a medium level of LD (*r*^2^ = 0.25), while the others showed an extremely low level of LD (*r*^2^ = 0.01–0.12).

### 3.4. LD between 50K SNPs and Gene Polymorphisms

**Figure 2:**
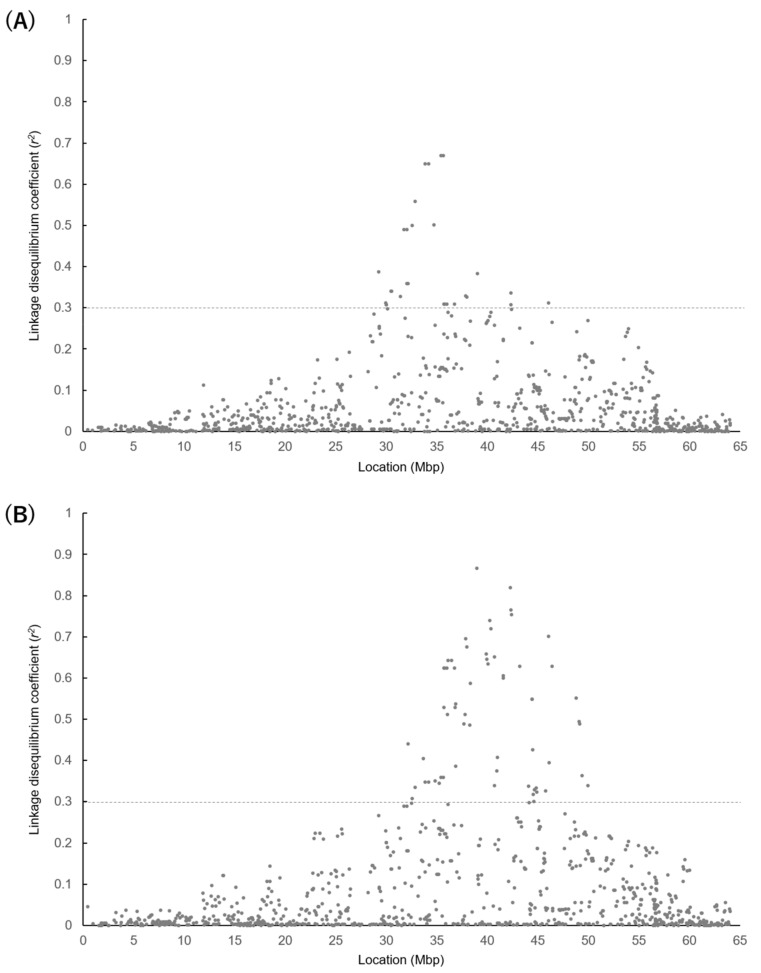
Linkage disequilibrium coefficients (*r*^2^) between 50K SNPs and *SREBP1* c.1065 + 83 (**A**) or *STARD3* c.1187 C > T (**B**) in Hyogo population (n = 200). *X*-axis represents SNP locations of 50K SNPs on BTA19 while *y*-axis represents *r*^2^-values.

**Figure 3:**
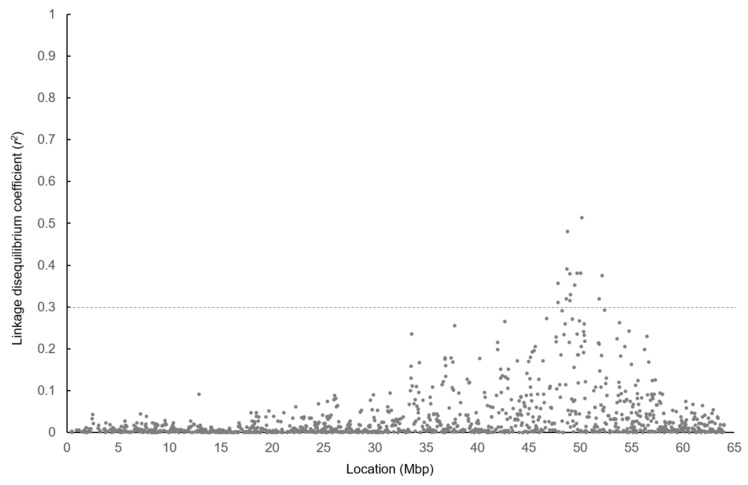
Linkage disequilibrium coefficients (*r*^2^) between 50K SNPs and *GH* c.379 C>G in Gifu population (n = 443). The *X*-axis represents SNP locations of 50K SNPs on BTA19 while the *y*-axis represents *r*^2^-values.

The LD coefficient (*r*^2^) between 50K SNPs and *SREBP1* c.1065 + 83 or *STARD3* c.1187 C > T in the Hyogo population can be seen in Figure 2, and the LD coefficient between the 50K SNPs and *GH* c.379 C > G in the Gifu population can be seen in Figure 3. Additionally, they provide an estimate of the range of the candidate region for the responsible gene of the QTL. An *r*^2^ > 0.30 was found for the *SREBP1* and *STARD3* polymorphisms from 29.2 to 46.4 Mbp and from 32.2 to 49.9 Mbp, respectively, in the Hyogo population. Among the 50K SNPs, UA-IFASA-6016 showed the maximum LD coefficient for the *STARD3* polymorphism (*r*^2^ = 0.87). However, the LD (*r*^2^ > 0.30) for the *GH* polymorphism was observed in a relatively narrow range of about 47.8–52.1 Mbp in the Gifu population. Moreover, the maximum LD coefficient was not very high (*r*^2^ = 0.51).

## 4. Discussion

This study was focused on five gene polymorphisms in order to estimate the QTL region for the percentage of C18:1 on BTA19 in Japanese Black cattle. First, the association was verified between the five polymorphisms and the percentage of C18:1 in two Japanese Black cattle populations (i.e., the Hyogo and Gifu populations). Previous studies have reported that *FASN* polymorphisms are significantly associated with fatty acid composition in cattle populations [3,4,5,6], whereas *FASN* polymorphisms demonstrated no effect on the percentage of C18:1 in the Hyogo population. This finding could be caused by the fixing of alleles in both SNPs, which is corroborated by the finding that the minor allele frequencies of *FASN* g.841G > C and g.16024 A > G were 0.00 in 44 animals and 0.10 in 441 animals, respectively. Moreover, *SCD* gene polymorphism, which is another polymorphism that is likely responsible for fatty acid composition, has been found to be also almost fixed in Japanese Black cattle from the Hyogo Prefecture [20]. Although the beef produced in Hyogo is known for its quality, and these results suggest that the Hyogo population had been selected for fatty acid composition, this population has never been directly selected for it. These polymorphisms might have been indirectly selected under selection pressure. The Hyogo population is also known for a high level of inbreeding due to the uniquely closed breeding without any migrations from other populations. In fact, Honda et al. [23] reported that the average inbreeding coefficients for the Japanese Black cattle population of the Hyogo Prefecture in 1960, 1970, 1980, and 1998 were 0.03, 0.05, 0.08, 0.14, and 0.19, respectively. The recent inbreeding level was higher than the inbreeding level in the entire Japanese Black cattle population during the same period (<0.06) [24]. This is because the Hyogo population has been almost closed to the others and intensive inbreeding has been conducted for the improvement of beef quality. Therefore, the alleles in a genomic region associated with beef quality were fixed because composition of fatty acids partly determines the total quality. In conclusion, the fixing of *FASN* and *SCD* polymorphisms could have been caused by selection for beef quality in the Hyogo population. This might also provide evidence for the large effects of these polymorphisms on beef quality.

However, the *STARD3* and *SREBP1* polymorphisms were significantly associated with the percentage of C18:1 in the Hyogo population and were in moderate LD (*r*^2^ = 0.29). Previous studies reported that polymorphisms in relatively low LD (*r*^2^ = 0.10–0.28) show similar effects [25,26]. Considering this, the polymorphisms within the *STARD3* (position: 35,234,637 bp to 35,250,672 bp) and *SREBP1* (position: 40,664,260 bp to 40,688,948 bp) genes seem to be included in a single candidate region for a common responsible gene. Therefore, both polymorphisms are significantly associated with C18:1 in the Hyogo population. As the molecular mechanisms that affect fatty acid composition by these polymorphisms have not been demonstrated yet, another polymorphism might be responsible for the QTLs near the *STARD3* and *SREBP1* genes. Additionally, a more accurate range of the candidate region for a responsible gene of the QTL was estimated by calculating an LD coefficient (*r*^2^) between 50K SNPs and *STARD3* or *SREBP1* polymorphisms. As *STARD3* and *SREBP1* polymorphisms showed a strong association with the percentage of C18:1, these polymorphisms are in relatively high LD with a responsible polymorphism (at least *r*^2^ = 0.30). Hence, we focused on SNPs that showed an *r*^2^ of more than 0.30 with both *STARD3* and *SREBP1* polymorphisms, and we found that the SNPs were located from 32.2 to 46.4 Mbp. The responsible polymorphism for the QTL would be located within the candidate region.

In the Gifu population, *FASN* and *GH* polymorphisms were significantly associated with the percentage of C18:1. The two *FASN* polymorphisms were in moderate LD (*r*^2^ = 0.25) with each other. Moreover, a previous study [5] suggested that the SNP g.841 G > C is responsible for fatty acid composition in the Japanese Black cattle population in Gifu Prefecture. In this previous study, g.841 G > C and g.16024 A > G were genotyped in order to compare their effects on fatty acid composition, and both SNPs were significantly associated with fatty acid composition. However, the SNP g.16024 A > G exhibited little or no effects on fatty acid composition when its effect was analyzed using an analytical model including the effect of the g.841 G > C genotype as a co-factor. Considering these results, g.841 G > C is responsible for the percentage of C18:1 in the Gifu population, and the effect of g.16024 A > G was observed due to LD with g.841 G > C.

The *GH* polymorphism showed extremely low LD with g.841 G > C. Matsuhashi et al. [9] also reported that the *GH* polymorphism was significantly associated with the percentage of C18:1 in a Japanese Black cattle population that was bred during a different period in Gifu Prefecture. Although the functional mechanism for the regulation of fatty acid composition by the *GH* gene has not been elucidated, a responsible polymorphism is likely to be located within or near the gene. The range of the candidate region for a responsible gene of the QTL was additionally determined based on the LD coefficient (*r*^2^) between the 50K SNPs and *GH* polymorphism. Similar to the QTL in the Hyogo population, we focused on SNPs with an *r*^2^ of more than 0.30 with *GH* polymorphism. As the SNPs were located from 47.8 to 52.1 Mbp, it would be possible to identify a responsible gene and polymorphism for the percentage of C18:1 by further investigating the candidate region.

Additionally, another difference between the Gifu and Hyogo populations was detected. *SREBP1* and *STARD3* polymorphisms were not significantly associated with the percentage of C18:1 in the Gifu population, while they exhibited a large effect on the trait in the Hyogo population. Therefore, the QTL might not be located near the *SREBP1* and *STARD3* genes in the Gifu population. However, *SREBP1* showed a value close to significant association with the percentage of C18:1 (*p* = 0.06). Additionally, a responsible polymorphism for the QTL could be relatively distant from the *SREBP1* and *STARD3* polymorphisms, as the candidate region identified in the Hyogo population exhibited a wide range. Considering these facts, the difference between populations might be due to lower LD between these polymorphisms and a responsible polymorphism in the Gifu population rather than in the Hyogo population. Additionally, the *SREBP1* polymorphism was previously found to be significantly associated with fatty acid composition in other populations of Japanese Black cattle and foreign cattle breeds in previous studies [13,27,28]. Therefore, it is necessary to conduct further verification for the existence of a QTL near the *SREBP1* and *STARD3* genes using other polymorphisms in the Gifu population.

**Figure 4:**
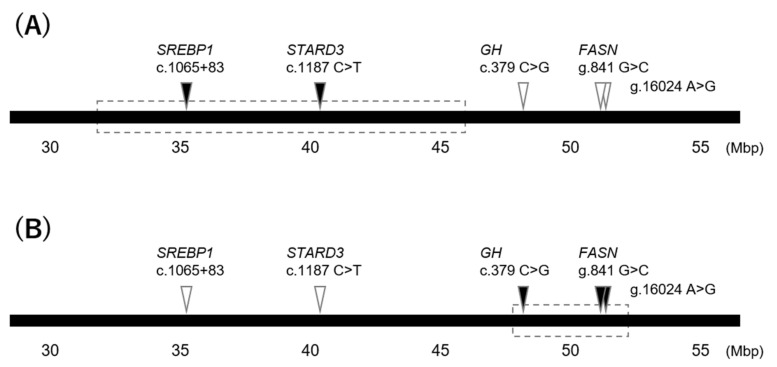
Estimated QTL positions on BTA19 in each population. The closed bar indicates a part of bovine chromosome 19 with approximate locations (Mbp). The dotted boxes indicate positions of estimated QTL in Hyogo (**A**) and Gifu (**B**) populations. The inverted triangles indicate positions of the polymorphisms which were significantly associated with C18:1 percentage (closed) or not associated (opened).

In the current study, the QTL for the percentage of C18:1 near the *FASN* gene was verified. Additionally, we detected a candidate region for the percentage of C18:1 including the *SREBP1* and *STARD3* polymorphisms in the Hyogo population and the *GH* polymorphism in the Gifu population (Figure 4). In Hyogo population, the allele of *GH* polymorphism was almost fixed and, therefore, the QTL near the *GH* gene was not detected in this study. Meanwhile, the QTL near *SREBP1* and *STARD3* genes was also not detected, probably due to extremely low level of LD with a responsible polymorphism in the Gifu population. In conclusion, the QTL position depends on populations because of various factors such as allele frequency and LD. The current study, moreover, succeeded in dividing each QTL region based on LD coefficients. A novel responsible gene and polymorphism for the percentage of C18:1 would be identified within each region by further investigations. Although this was the first study to verify the effects and LD of previously reported polymorphisms, which were investigated using different populations, our results suggest that verification using the same population reveals a more accurate number and position of the QTL. In the past few decades, several studies have reported significant associations between polymorphisms and economic traits in cattle. However, most of the responsible polymorphisms have not been elucidated yet. Further research is required, using a verification approach similar to that of the current study, in order to more clearly define the candidate regions and contribute to an efficient search for responsible genes and polymorphisms.

## Figures and Tables

**Table 1:** Summary statistics of the two Japanese Black populations.

		Hyogo Population	Gifu Population
n	Total	441	443
	steers/heifers	352/89	443/0
Sires		7	57
Age at slaughter (months)	Mean ± SD	31.69 ± 1.24	28.32 ± 1.10
	Max	35.87	34.62
	Min	28.44	25.74
C18:1 percentage (%)	Mean ± SD	54.76 ± 3.01	51.92 ± 3.29
	Max	64.01	60.54
	Min	45.45	43.67

**Table 2:** Polymorphisms located on BTA19 and significantly associated with fatty acid composition in Japanese Black cattle in previous studies.

Location	ID	Gene	Position	Polymorphism	Amino Acid	Reference
35,244,514	rs133958066	*SREBP1*	Intron 5	c.1065+83 (84bp indel)	-	[7]
40,687,937	rs134877666	*STARD3*	Exon 14	c.1187 C > T	S396L	[8]
48,768,916	rs41923484	*GH*	Exon 5	c.379 C > G	L127V	[21]
51,384,984	rs41920005	*FASN*	Exon 1	g.841 G>C	-	[5]
51,400,139	rs480320793	*FASN*	Exon 34	g.16024 A>G	T1950A	[3]

Location, location of polymorphisms on BTA19 based on the genome reference, Bos_taurus_UMD_3.1.1; Position, position of polymorphism for each gene; Amino acid, substitution of amino acids caused by the polymorphism.

**Table 3:** Protocol summary for genotyping by PCR-RFLP or TaqMan method.

Gene Marker	Method	Sequence (from 5′ to 3′)	AT	PL	RE	Reference
*SREBP1* c.1065 + 83	PCR	F: CCA CAA CGC CAT CGA GAA ACG CTA C	60	348/432	-	[7]
		R: GGC CTT CCC TGA CCA CCC AAC TTA G				
*STARD3* c.1187 C > T	PCR-RFLP	F: AGG AGG ATT TGA GCA CCC CAT	60	351	*Msc*I	[8]
		R: CAA GGT CAC ACA GCA CAC TCC				
*GH* c.379 C > G	PCR-RFLP	F: CTT AGC CAG GAG AAT GCA CG	66	605	*Alw*NI	-
		R: ATG CCT GCT ATT GTC TTC CC				
	Taq-Man	F: CAA ATT TGT CAT AGG TCT GCT TG	64	228	-	-
		R: CCC TCT TTC TAG CAG TCC AG				
		P1: FAM- CAT CTT CCA GCT CCT GCC A -BHQ				
		P2: HEX- CAT CTT CCA CCT CCT GCC A -BHQ				
*FASN* g.841 G > C	Taq-Man	F: ACA CTC CAT CCT CGC TC	60	102	-	[5]
		R: TCC CGA CTC GCA ACT TC				
		P1: FAM- ACA GCC GCC CGC G -BHQ				
		P2: HEX- ACA GCC CCC CGC GC -BHQ				

AT, annealing temperature (°C); PL, PCR product length (bp); RE restriction enzyme.

**Table 4:** Genotype and allele frequencies for the five polymorphisms and their association with the percentage of C18:1 in the Hyogo population (n = 441).

Polymorphism	A Allele	B Allele	Genotype Frequency			Allele Frequency		*p*-Value	Means ± SE of C18:1		
			AA	AB	BB	A	B		AA	AB	BB
*SREBP1* c.1065 + 83	L	S	210	180	51	0.79	0.21	4.00 × 10^−4^	55.28 ± 0.20	54.56 ± 0.21	53.32 ± 0.47
*STARD3* c.1187 C > T	C	T	141	206	94	0.55	0.45	1.68 × 10^−5^	55.88 ± 0.25	54.65 ± 0.19	53.29 ± 0.29
*GH* c.379 C > G	G	C	48	0	0	1.00	0.00	-	-	-	-
*FASN* g.841 G > C	G	C	44	0	0	1.00	0.00	-	-	-	-
*FASN* g.16024 A > G	A	G	360	73	8	0.90	0.10	0.614	54.63 ± 0.16	55.33 ± 0.34	55.09 ± 1.22

*p*-value: association was analyzed by analysis of variance.

**Table 5:** Genotype and allele frequencies for the five polymorphisms and their association with the percentage of C18:1 in Gifu population (n = 443).

Polymorphism	A Allele	B Allele	Genotype Frequency			Allele Frequency		*p*-Value	Means ± SE of C18:1		
			AA	AB	BB	A	B		AA	AB	BB
*SREBP1* c.1065 + 83	L	S	159	218	66	0.61	0.39	0.060	52.06 ± 0.26	52.09 ± 0.22	51.02 ± 0.37
*STARD3* c.1187 C > T	C	T	110	227	106	0.51	0.49	0.143	51.60 ± 0.33	51.83 ± 0.21	52.42 ± 0.31
*GH* c.379 C > G	G	C	189	208	46	0.66	0.34	4.00 × 10^−3^	52.58 ± 0.23	51.42 ± 0.24	51.46 ± 0.42
*FASN* g.841 G > C	G	C	362	78	3	0.91	0.09	8.24 × 10^−5^	52.23 ± 0.17	50.44 ± 0.37	52.79 ± 3.65
*FASN* g.16024 A > G	A	G	305	126	12	0.83	0.17	1.00 × 10^−3^	52.25 ± 0.19	51.25 ± 0.28	50.53 ± 1.03

*p*-value: association was analyzed by analysis of variance.

## Data Availability

Not applicable.
